# Treatment Efficacy of Immune Checkpoint Inhibitors for Patients with Advanced or Metastatic Colorectal Cancer: A Systematic Review and Meta-Analysis

**DOI:** 10.3390/jcm10163599

**Published:** 2021-08-16

**Authors:** Junhee Pyo, Hyo-Jung Park

**Affiliations:** 1 Asan Medical Center, Department of Biomedical Engineering, College of Medicine, University of Ulsan, Seoul 05505, Korea; stdpjh@mail.ulsan.ac.kr; 2 Asan Medical Center, Department of Radiology and Research Institute of Radiology, College of Medicine, University of Ulsan, Seoul 05505, Korea

**Keywords:** colorectal cancer, immune checkpoint, microsatellite instability, systematic review, treatment response assessment

## Abstract

The treatment efficacy of immune checkpoint inhibitors (ICIs) in colorectal cancer (CRC) has been reported heterogeneously across clinical trials. We conducted a systematic review and meta-analysis to evaluate the efficacy of ICIs in patients with advanced/metastatic CRC. Ovid-Medline was searched to identify clinical trials providing the efficacy outcomes of overall response rate (ORR) or disease control rate (DCR). The pooled ORR and DCR were estimated across all studies and subgroups. Meta-regression was performed to find the influencing factors for treatment efficacy. A total of thirty studies (1870 patients) were eligible. The overall ORR and DCR were 20.1% and 58.5%, respectively, but these results were heterogeneous across studies. Multivariate meta-regression revealed that microsatellite phenotype (odds ratio of MSI-H/dMMR versus MSS/pMMR: 1.67, *p* < 0.001) and drug regimen (odds ratio of monotherapy versus combination therapy: 1.07, *p* = 0.019) were the source of heterogeneity and also significantly influenced factors for the efficacy of the treatment. Although the efficacy of ICIs as a first-line therapy was higher than that of ICIs as the second- or more-line therapy (ORR: 51.5% vs. 13.4%, DCR: 85% vs. 49.5%), multivariate regression showed that the line of therapy was not a significant factor for the treatment efficacy. Our study suggests that the microsatellite phenotype and drug regimen, rather than the line of treatment, are the primary factors influencing the treatment response among advanced/metastatic CRC patients treated with an ICI-based regimen.

## 1. Introduction

Colorectal cancer (CRC) remains a leading cause of cancer-related death worldwide, with a 5-year survival rate for 14.3% for patients with metastatic CRC [1,2]. In addition to the development of chemotherapies, biologics, and targeted therapies, the introduction of immunotherapy with immune checkpoint inhibitors (ICIs) including anti-programmed death 1 (PD-1), anti-programmed death-ligand 1 (PD-L1), or anti- cytotoxic T-lymphocyte antigen 4 (CTLA-4) has led to changes in the management of metastatic CRC, thereby leading to meaningful improvements in survival and radiological response [3].

The activity of ICIs has been noted for CRC with microsatellite instability (MSI) or mismatch repair deficiency (dMMR), which leads to the high mutational load and upregulated expression of multiple immune checkpoints. MSI-H/dMMR CRCs display a good response to ICI, owing to their hyper-mutated nature. Nivolumab (anti PD-1) or pembrolizumab (anti PD-1) monotherapy as well as the combination of nivolumab and ipilimumab (anti CTLA-4 agent) were found to result in a good treatment response and improved survival outcomes among patients with refractory MSI-H/dMMR metastatic CRC [4,5,6] and were approved by the Food and Drug Administration (FDA) for the treatment of MSI-H/dMMR metastatic CRC that experienced disease progression following chemotherapy. Not only as a second- or more-line therapy, pembrolizumab was also demonstrated to be superior to the conventional chemotherapy when used as the first-line treatment in the phase III KEYNOTE 177 trial [7], which led to the FDA approval of pembrolizumab as the first-line treatment for patients with unresectable or metastatic MSI-H/dMMR CRC. Since then, many trials have been conducted or are ongoing to investigate the treatment effect of various ICI-based regimens in patients with untreated MSI-H/dMMR metastatic CRC [8,9,10]. However, no attempt has been made to generate a systematic summary on the overall treatment efficacy of ICI treatment on those patients yet.

On the other hand, microsatellite-stable (MSS) or proficient MMR (pMMR) CRC displays a low mutational load, and many ICI treatment results have been disappointing for MSS/pMMR CRC [11,12,13]. However, conflict results do exist regarding the treatment efficacy of combination therapy with ICIs and other agents with different mechanisms of action [14,15,16,17]. The treatment efficacy of an ICI-based treatment for patients with MSS/pMMR CRC remains to be elucidated.

With the anticipated availability of several ICI-based regimens for the management of advanced/metastatic CRC, how to plan a specific treatment course with ICI agents is an important question. Rapidly accumulating evidence from trials evaluating the treatment efficacy of ICIs warrants a summary, which would allow a more evidence-based management of patients with advanced/metastatic CRC. The aim of this study was to conduct a systematic review and meta-analysis to evaluate the treatment efficacy of ICIs for patients with advanced/metastatic CRC using clinical trial data.

## 2. Materials and Methods

### 2.1. Literature Search 

The literature review and meta-analysis were conducted following the Preferred Reporting Items for Systematic Reviews and Meta-Analyses (PRISMA) guidelines. A systematic computerized search of Ovid-Medline and EMBASE databases was conducted to identify relevant studies published before November 2020, with restriction to articles written in English. The following search terms were used: ((“response”[Title/Abstract] OR “response rate”[Title/Abstract] OR “overall response rate”[Title/Abstract] OR “ORR”[Title/Abstract] OR “control rate”[Title/Abstract] OR “DCR”[Title/Abstract]) AND ((“colorectal cancer”[Title/Abstract] OR “colon cancer”[Title/Abstract] OR “rectal cancer”[Title/Abstract]) AND (“immune checkpoint inhibitor”[Title/Abstract] OR “checkpoint inhibitor”[Title/Abstract] OR “checkpoint”[Title/Abstract] OR “PD-1”[Title/Abstract] OR “PD-L1”[Title/Abstract] OR “CTLA-4”[Title/Abstract] OR “pembrolizumab”[Title/Abstract] OR “nivolumab”[Title/Abstract] OR “atezolizumab”[Title/Abstract] OR “avelumab”[Title/Abstract] OR “durvalumab”[Title/Abstract])))). We also reviewed the bibliographies of the selected studies to ensure that other eligible articles were included.

### 2.2. Eligibility Criteria and Quality Assessment 

Studies were eligible for inclusion if patients with CRC were treated with ICIs and were evaluated based on the efficacy outcome measures of overall response rate (ORR) or disease control rate (DCR) according to Response Evaluation Criteria for Solid Tumors (RECIST) v1.1 [18]. ORR, a direct measure of tumoricidal activity of treatment, is defined as the proportion of patients who achieve a complete response (CR) or partial response (PR) per RECIST v1.1. DCR, an index that is used to measure the tumoristatic effects of treatment, is defined as the proportion of patients who achieved a CR, PR, and stable disease (SD) per RECIST v1.1. Studies were excluded if they were animal/in vitro studies, reviews and editorials, case reports, study protocols, conference proceedings, included no CRC patients, included no ICI use, and included no interest of the study purpose. The risk of bias and methodologic quality were evaluated using the Cochrane Risk of Bias 2.0 [19] for randomized clinical trials and the Newcastle–Ottawa scale [20] for nonrandomized trials.

### 2.3. Data Extraction and Synthesis 

There were two reviewers who independently reviewed the articles based on a standardized protocol; any disagreement was resolved in a meeting where a consensus was established. The following information was extracted from the eligible studies: (a) study characteristics: authors, publication year, trial phase, and enrollment periods; (b) patient characteristics: number of patients, tumor stage, microsatellite phenotype, drug type, and treatment line; and (c) study outcomes: number of overall response and diseases controlled. To explore the treatment efficacy as measured by ORR and DCR, pooled ORR and DCR were adopted as metameters for our data synthesis. 

### 2.4. Statistical Analysis 

DerSimonian–Laird random-effect models were constructed to synthesize the pooled ORR and DCR percentage with 95% confidence intervals (CIs) [21]. To assess publication bias, we plotted funnel plots and conducted Begg’s test to detect asymmetry [22]. To investigate the heterogeneity, we performed Cochran’s Q test and I^2^ statistics, with significance identified if the I^2^ statistics were greater than 50% and if the *p*-value of Cochran’s Q test was less than 0.10 [23]. 

Due to the heterogeneity of the studies included, random effects models were used to estimate the pooled effect. Univariate and multivariate meta-regression analyses were conducted to explore the influencing factors for treatment efficacy and revealed the source of heterogeneity. In the meta-regression analysis, the Knapp and Hartung adjustments were applied, which are typically adopted in the meta-regression mixed effect model to control the type 1 error rate of 0.05; these values were reported as multiplicity-adjusted *p*-value and a 95% CI [24,25,26]. Additionally, we performed the leave-one-out sensitivity analysis by iteratively removing one study at a time to verify the dependency of the result on a single study. Subgroup analysis was performed by calculating the pooled ORR and DCR according to each category of microsatellite phenotype and drug regimen (Category 1, monotherapy and MSI-H/dMMR; Category 2, monotherapy and MSS/pMMR; Category 3, combination therapy and MSI-H/dMMR; and Category 4, combination therapy and MSS/pMMR) in patients treated with ICIs as second- or more-line therapy. R version 4.0.3 (R foundation for Statistical Computing, Vienna, Austria) was used for analysis with the ”meta” packages. 

## 3. Results

### 3.1. Literature Search

**Figure 1:**
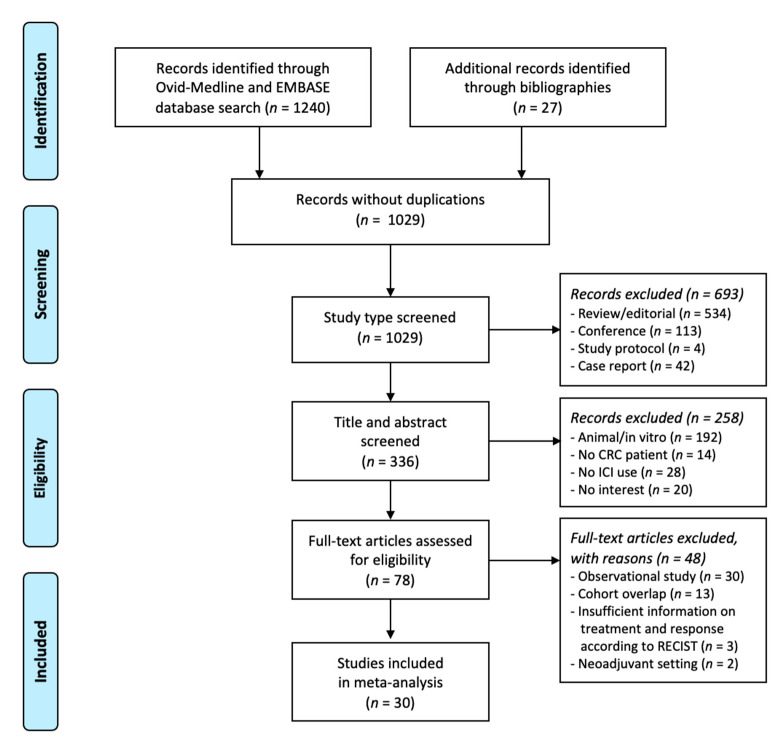
Flow diagram of the study selection process. RECIST, Response Evaluation Criteria for Solid Tumors.

Following an electronic search and a review of bibliographies, 10,290 studies were identified. Of these, 693 were excluded after a review of the study type; reviews/editorials, conference presentations, study protocols, and case reports were excluded. A review of the title and the abstract of remaining studies was conducted. Thereafter, 78 studies were retained after excluding animal/in vitro studies, studies without CRC patients, studies with no ICI use, and studies with no interest of the study purpose. After a full text review of the 78 studies, 48 studies were excluded: observational studies (n = 30); cohort overlap (n = 13); insufficient information provided on treatment response according to RECIST v1.1 (n = 3); and neoadjuvant setting (n = 2). Finally, a total of 30 eligible clinical trials were included in this systematic review and meta-analysis [5,6,7,8,9,10,14,15,16,17,27,28,29,30,31,32,33,34,35,36,37,38,39,40,41,42,43,44,45,46] (Figure 1). 

### 3.2. Study Characteristics

Table 1 lists the characteristics of the 30 included clinical trials with subgroup data. The study population included patients with advanced or metastatic CRC. 

There were 18 phase 2 studies, ten phase 1 studies, and two phase 3 studies. ICIs were administered as a monotherapy in ten studies or as combination therapy with other ICIs or other agent(s) with different mechanisms of action in 19 studies. There was one study that included both monotherapy and combination therapy with ICIs [31]. Patients were treated with ICIs as the first-line therapy in six studies and as the second- or more-line therapy in 24 studies. The microsatellite phenotype of CRC was MSI-H/dMMR in six studies, MSS/pMMR in seven studies, and MSI-H/dMMR or POLE mutation in one study. There were six studies that included both MSI-H/dMMR and MSS/pMMR tumors. There were ten studies that did not report the microsatellite phenotype of CRC. As an efficacy measure, the ORR and DCR were extracted for each trial. 

**Figure 2:**
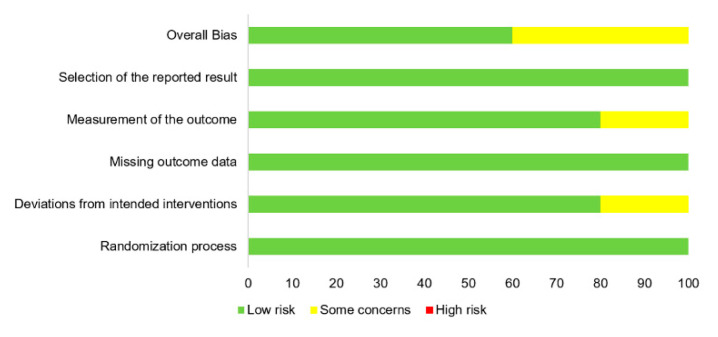
Quality assessment of the five randomized clinical trials.

Among the five randomized clinical trials [7,8,31,37,45], the risk of bias based on the Cochrane Risk of Bias 2.0 tool was identified to be low for the three studies, and there were some concerns for overall bias in two studies (Figure 2). For 25 non-randomized trials, the Newcastle–Ottawa scale score ranged from 6 to 8 points, indicating the high quality of the included studies (Table 2).

### 3.3. Efficacy Endoints: ORR and DCR

**Figure 3:**
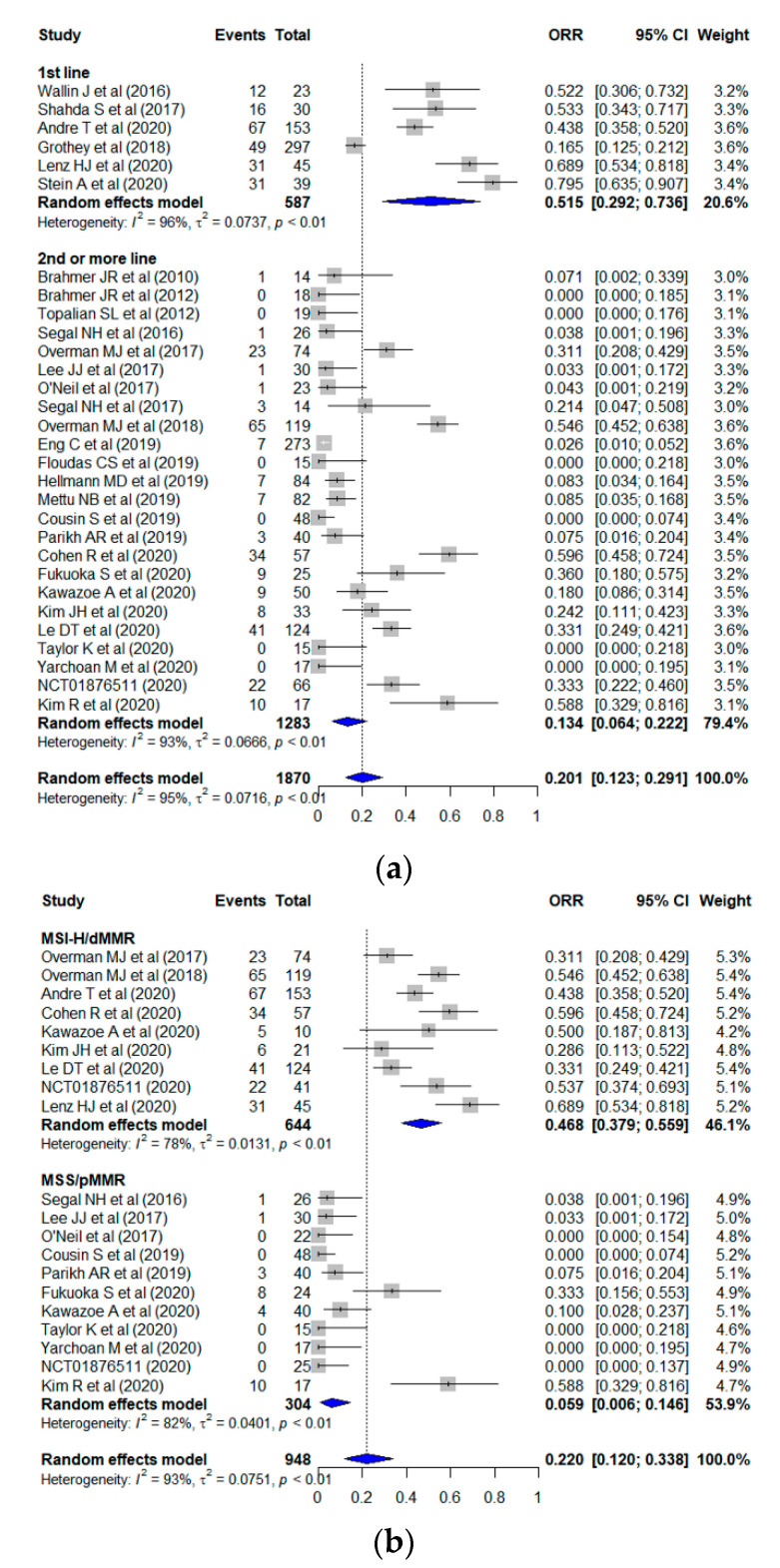

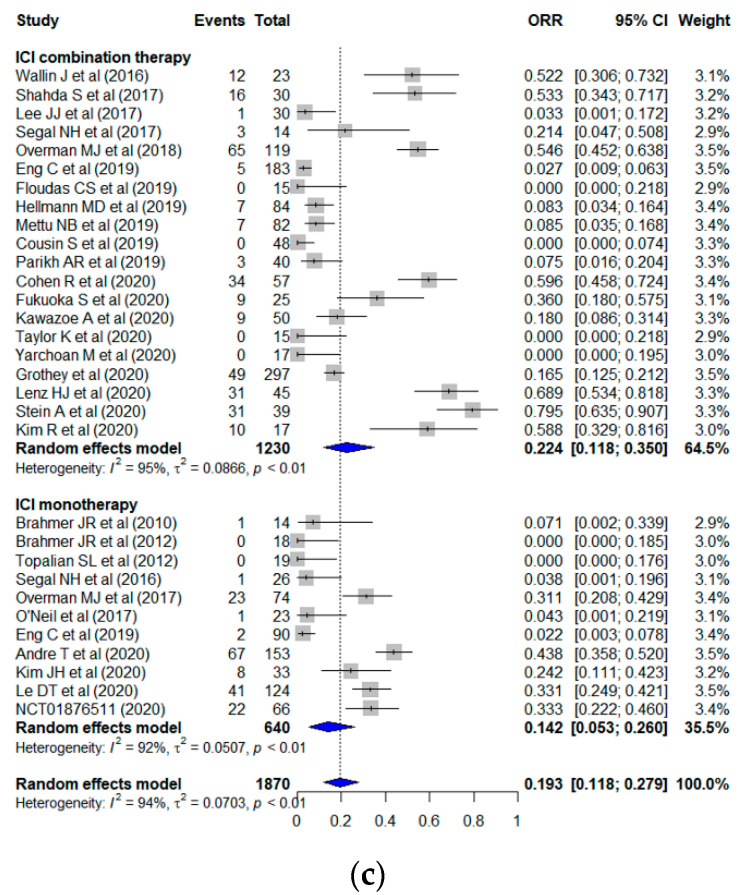
Forest plots displaying the pooled ORR according to (**a**) treatment line, (**b**) microsatellite phenotype, and (**c**) drug regimen. The blue quadrilaterial indicates the pooled incidence and its 95% confidence interval.

The pooled ORR from all of the studies was 20.1% (95% CI, 12.3–29.1%) (Figure 3a). The leave-one-out sensitivity analysis revealed that the pooled ORR ranged from 18.3% to 21.3% when each study was excluded. The funnel plot revealed no publication bias across the studies (*p* = 0.475) (Figure S1). The pooled estimates of ORR in the subgroups are shown in Figure 3a–c. The pooled ORR among patients treated with ICIs as the first-line and second- or more-line was 51.5% (95% CI, 29.2–73.6%) and 13.4% (95% CI, 6.4–22.2%), respectively; significant difference (*p* = 0.003) was observed between the two values. According to the microsatellite phenotype, the pooled ORR was 46.8% (95% CI, 37.9–55.9%) in MSI-H/dMMR tumors and 5.9% (95% CI, 0.6–14.6%) in MSS/pMMR tumors (*p* < 0.001). Regarding drug regimen, the pooled ORR of patients treated with the ICI monotherapy was 14.2% (95% CI, 5.3–26.0%); which was slightly lower than that of patients treated with the combination therapy (22.4%; 95% CI, 11.8–35.0%) (*p* = 0.332). Heterogeneity was observed for all of the studies (I^2^ = 94%) and each subgroup (first line, I^2^ = 96%; second- or more-line, I^2^ = 93%; MSI-H/dMMR, I^2^ = 83%; MSS/pMMR, I^2^ = 94%; ICI monotherapy, I^2^ = 92%; combination therapy, I^2^ = 95%).

**Figure 4:**
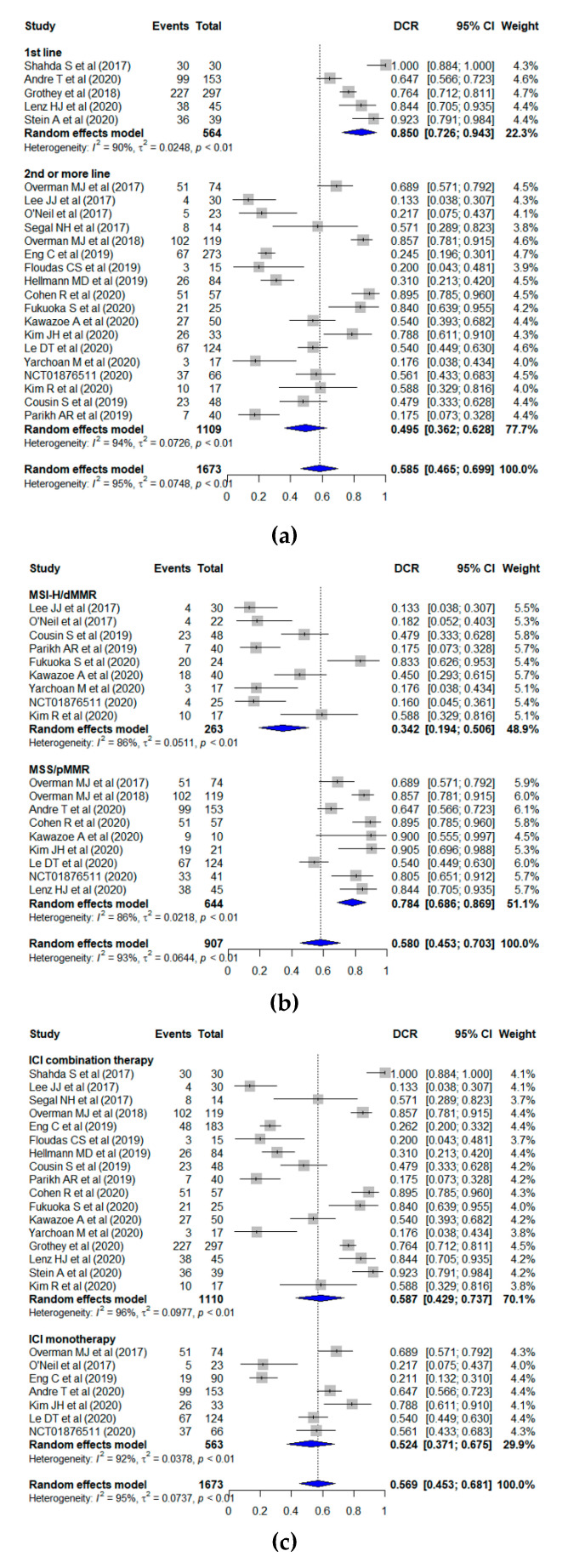
Forest plots displaying the pooled DCR according to (**a**) treatment line, (**b**) microsatellite phenotype, and (**c**) drug regimen.

The pooled DCR from all of the studies was 58.5% (95% CI, 46.5–70.0%) (Figure 4a). When the leave-one-out sensitivity analysis was conducted, the pooled DCR was found to range from 55.8% to 60.7%. The funnel plot showed no publication bias across studies based on the Begg’s test (*p* = 0.508), which was conducted to test the funnel plot asymmetry (Figure S2). The pooled estimates of DCR in the subgroups are shown in Figure 4a–c. The pooled DCR was 85.0% (95% CI, 72.6–94.3%) for patients treated with ICI as the first-line therapy and 49.5% (95% CI, 36.2–62.8%) for those treated with ICI as the second- or more-line therapy (*p* = 0.006). According to the microsatellite phenotype, the pooled DCR was 78.4% (95% CI, 68.6–86.9%) in tumors with MSI-H/dMMR and 34.2% (95% CI, 19.4–50.6%) in tumors with MSS/pMMR (*p* < 0.001). Regarding drug regimen, the pooled DCR of the patients treated with the ICI monotherapy was 52.4% (95% CI, 37.1–67.5%) and that of the patients treated with the combination therapy was 58.7% (95% CI, 42.9–73.7%); no significant difference was identified (*p* = 0.632). Heterogeneity was observed for all studies (I^2^ = 94%) and each treatment line group (first-line, I^2^ = 90%; second- or more-line, I^2^ = 94%), microsatellite phenotype (MSI-H/dMMR, I^2^ = 86%; MSS/pMMR, I^2^ = 86%), and drug regimen (ICI monotherapy, I^2^ = 92%; combination therapy, I^2^ = 96%). 

In three studies in which patients with MSS/pMMR tumors were treated with ICI combined with regorafenib as the second- or more-line therapy [14,16,17], the DCR ranged from 47.9% to 83.3%, while the other regimens ranged from 13.3% to 45.0%. Among the three studies with ICI plus regorafenib, two studies performed by Fukuoka et al. [14] and Kim et al. [16] also showed markedly higher ORR (33.3% and 58.8%, respectively) than any other regimen (range, 0–07.5%).

### 3.4. Meta-Regression Analysis

We explored the influencing factors for the ORR and DCR by performing a meta-regression. In the univariate analysis, patients treated with ICI as the second- or more-line had a significantly lower ORR than those treated with ICI as the first-line treatment (Odds ratio [OR], 0.67; 95% CI, 0.52–0.86). Patients with MSI-H/dMMR tumors had significantly higher ORR (OR, 1.61; 95% CI, 1.37–1.90) than those with MSS/pMMR tumors. Slightly higher ORR was observed for patients treated with ICI combination therapy than for those treated with the ICI monotherapy (OR, 1.11; 95% CI, 0.89–1.39). In the multivariate analysis with covariates of the treatment line, microsatellite phenotype and drug regimen, and microsatellite phenotype and drug regimen appeared to significantly influence ORR. The OR of patients with MSI-H/dMMR tumors was 1.67 (95% CI, 1.42–1.98) with patients having MSS/pMMR tumors as the reference, and the OR of patients with ICI combination therapy was 1.24 (95% CI, 1.02–1.49) with patients who received ICI monotherapy as the reference (Table 3). The treatment line showed no significant difference between the first-line and the second- or more-line (OR, 0.90; 95% CI, 0.71–1.15 with the first-line as the reference). 

The univariate and multivariate meta-regression analyses for DCR showed a similar trend to those for ORR. In the univariate analysis, the cohorts treated with ICIs as the second- or more-line treatment had significantly lower DCR than those treated with ICIs as the first-line treatment (OR, 0.68; 95% CI, 0.52–0.88). Patients with MSI-H/dMMR tumors had significantly higher DCR (OR, 1.57; 95% CI, 1.29–1.91) than those with MSS/pMMR tumors. Patients treated with ICI combination therapy had slightly higher DCR than those treated with ICI monotherapy (OR, 1.07; 95% CI, 0.81–1.39). In the multivariate analysis, the microsatellite phenotype and the drug regimen appeared to be the significant factor influencing DCR (OR, 1.72 (95% CI, 1.41–2.10) for MSI-H/dMMR; OR, 1.24 (95% CI, 1.02–1.49) for ICI combination therapy) (Table 3).

### 3.5. Subgroup Analysis

**Figure 5:**
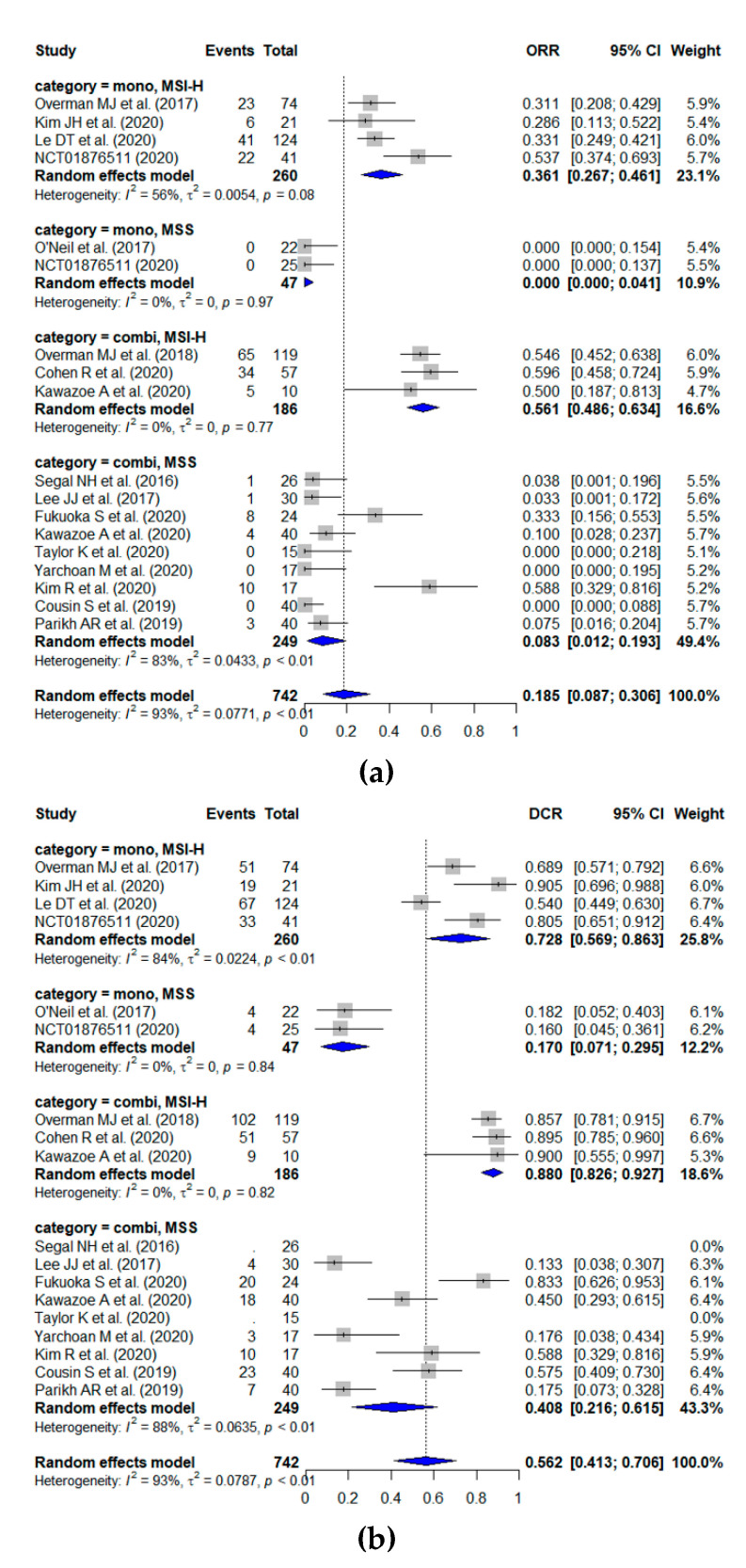
Forest plots of the (**a**) pooled ORR and (**b**) DCR of each subgroup according to the microsatellite phenotype and drug regimen in patients treated with ICIs as second- or more-line therapy.

The pooled incidence of ORR and DCR in subgroups according to the microsatellite phenotype and drug regimen in patients treated with ICIs as second- or more-line therapy are provided in Table 4 with forest plots in Figure 5. Regarding ORR, the pooled ORR of Category 3 (combination therapy, MSI-H) was the highest (56.0%), followed by Category 1 (monotherapy, MSI-H; 36.2%), Category 4 (combination therapy, MSS; 10.9%), and Category 2 (monotherapy, MSS; 2.0%). With the exception of Category 2 versus Category 4 (*p* = 0.318), all inter-category comparisons showed significant differences (*p* ≤ 0.017). As for DCR, the same trend was observed as in ORR. The pooled DCR was the highest in Category 3 (combination therapy, MSI-H; 87.3%), followed by Category 1 (monotherapy, MSI-H; 73.0%), Category 4 (combination therapy, MSS; 41.5%), and Category 2 (monotherapy, MSS; 17.0%). With the exception of Category 1 vs. Category 3 (*p* = 0.32) and Category 2 vs. Category 4 (*p* = 0.142), all of the comparisons showed significant differences (*p* ≤ 0.011). Heterogeneity decreased in Category 1 and cCtegory 4 in pooling the of the ORR (I^2^ = 56% and 83%, respectively) and the DCR (I^2^ = 84% and 88%, respectively), and there was no heterogeneity in the pooling of both estimates in Category 2 and Category 3 (I^2^ = 0%).

## 4. Discussion

In this study, we evaluated the treatment efficacy of ICI-based therapy for patients with advanced/metastatic CRC using the data from 30 clincial trials. The findings of this meta-analysis indicate that the microsatellite phenotype of tumor and drug regimen significantly influence the treatment response of patients with advanced/metastatic CRC administered ICI. In MSS/pMMR tumors, a durable response was noted in the second- or more-line treatment when ICI was administered as part of a combination treatment. 

We used ORR and DCR as the primary endpoints of our analysis. Although survival time such as overall survival has been regarded as the most reliable metric for assessing the efficacy of anticancer treatment, overall survival has several drawbacks when used a primary endpoint in clinical trials; the sample size needs to large enough, a much longer follow-up is required than other endpoints as the time to event (i.e., death) is much longer, and the analysis may be much more confounded than other endpoints by the effect of salvage therapies used after disease progression [47]. Since the ORR and DCR are the most commonly used primary or secondary endpoints in clinical trials, we could summarize the results using these endpoints from the most clinical trials to acquire more generalizable summary estimates. 

Our study showed that the microsatellite phenotype significantly affected treatment efficacy, irrespective of the treatment line (i.e., first-line or second- or more-line) and drug regimen (i.e., ICI monotherapy or combination), as revealed by multivariate meta-regression analysis. Treatment efficacy was markedly higher in patients with MSI-H/dMMR tumors than in those with MSS/pMMR tumors. The pooled ORR and DCR of patients with MSI-H/dMMR tumors were 46.8% and 78.4%, respectively, while the pooled ORR and DCR of those with MSS/pMMR tumors were 5.9% and 34.2%, respectively. This finding is consistent with previous studies where a remarkable ICI efficacy was observed for patients with advanced or metastatic CRC and other solid tumors that are MSI-H or dMMR [5,48]. In addition to the MSI status, high mutational load (i.e., tumor mutational burden) and upregulated expression of PD-1/PD-L1 have been reported to be associated with an increased response rate to ICI treatment [49]. The association between MSI status and tumor mutation burden or the upregulated expression of multiple immune checkpoints has been suggested [50,51]. However, the relationship among these biomarkers is still unclear and needs further investigation. 

Regarding the drug regimen, ICI combination therapy (i.e., ICI with other ICI or non-immunotherapy drugs) resulted in a higher treatment response rate than ICI monotherapy. The pooled ORR and DCR of patients treated with the ICI combination therapy were 22.4% and 58.7%, respectively, while the pooled ORR and DCR of those treated with ICI monotherapy were 14.2% and 52.4%, respectively. Although the overall pooled ORR was not found to differ between ICI monotherapy and the combination therapy in univariate analysis, the combination therapy resulted in a significantly higher ORR than the monotherapy (OR, 1.21; 95% CI, 1.04–1.42) when stratified by the line of treatment and microsatellite phenotype. The combination therapy also resulted in a significantly higher DCR than monotherapy in the multivariate analysis (OR, 1.24; 95% CI, 1.02–1.49). 

The explicitly higher treatment efficacy of combination therapy including ICI and regorafenib [14,16,17] than the other regimens administered to patients with MSS/pMMR tumors administered ICI as a second- or more-line treatment is worth recognizing. The DCR of three studies on ICI plus rigorafenib ranged from 47.9% to 83.3%, while the DCR of other regimens ranged from 13.3% to 45.0%. Among the three studies, two studies performed by Fukuoka et al. [14] and Kim et al. [16] also showed markedly higher ORR (33.3% and 58.8%, respectively) than other regimens (range, 0–07.5%). This is surprising, considering the widespread concept of poor treatment efficacy and outcome in patients with MSS/pMMR tumors. Regorafenib is a multi-kinase inhibitor that targets a wide range of tyrosine kinases associated with oncogenesis, angiogenesis, and tumor microenvironment control [52]. The clinical potential of kinase inhibitors in combination with ICIs has been reported [53], which possibly results from the role of the kinase inhibitor in increasing tumor immunogenicity. Although low treatment efficacy has been reported [11,12,13], the long-lasting treatment response owing to the above combination therapies suggests the potential of ICI treatment for patients with MSS/pMMR CRC. The combination effect of ICI and other drugs with different mechanisms of action in MSS/pMMR CRC patients is an understudied topic [54] and is worth of further exploration. 

Based on the univariate meta-regression analysis, both ORR and DCR were significantly higher in patients who were administered ICI as the first-line treatment than in those who were administered ICI as the second- or more-line treatment. However, it was revealed not to be an influencing factor for the treatment efficacy in multivariate analysis. Such findings might be due to the more frequent use of the ICI combination therapy as the first-line treatment instead of as the second- or more-line treatment. However, the number of studies that administered combination therapy as the first-line treatment was small, which limits inference on the efficacy of first-line ICI treatment for patients with untreated advanced/metastatic CRC. Therefore, further trials are needed to determine the treatment efficacy according to the line of ICI treatment. The results of the ongoing trials of ICIs administered to patients with untreated metastatic CRC are highly anticipated. 

Our study had some limitations. First, the number of included trials with ICI as the first-line therapy was small. Nevertheless, all of the available information for the clinical trials performed to date has been included herein. The results of our study could serve as a basis for future studies when sufficent new data become available. Further, our findings will contribute to finding biologically meaningful combination therapies containing ICIs. Second, the included studies were highly heterogeneous, and this heterogeneity precluded us from acquiring a solid meta-analytic summary estimate of ORR and DCR across all 30 studies. When pooling all of the studies, the DerSimonian and Laird method was used, which is based on the normal approximation to result in mean value. However, this might be quite a strong assumption even if 30 studies were included, as the included studies were very heterogeneous. To reveal and explain the study heterogeneity and its reason for using a systematic approach, we explored the influencing factors for ORR and DCR by performing the meta-regression and subgroup analysis. Although the heterogeneity substantially decreased in subgroup analyses according to the microsatellite phenotype and drug regimen, significant heterogeneity still existed in the pooled ORR and DCR in Category 1 (monotherapy, MSI-H) and Category 4 (combination therapy, MSS; Table 4), and there could be other unknown source of heterogeneity. Third, our analysis to determine the cause of heterogeneity and influencing factors for treatment efficacy was limited by only useing the information that was available in the included studies (i.e., microsatellite phenotype, treatment line, and drug regimen (monotherapy vs. Combination)), and our finding that the microsatellite phenotype and drug regimen are the influencing factors of the treatment response in CRC patients after ICI treatment has been previously proposed. However, we validated those results through a comprehensive review and meta-analysis using the available clinical trial data to date by extracting all of the available and categorizable data from the included studies. Additionally, one of our findings that combination therapy with ICI and regorafenib as a second- or more-line treatment showed high efficacy in patients with MSS/pMMR tumors is worth noticing, which suggests the potential of ICI treatment for patients with MSS/pMMR CRC, and further research is required to figure out the nature of antitumoral response and an effective ICI regimen in MSS/pMMR CRC. Considering that the objectives of the systematic review and meta-analysis include obtaining more valid and generalizable values of the estimates of interest and identifying areas for further research, we believe our study is pertinent.

## 5. Conclusions

We evaluated the treatment efficacy of the ICI-based therapy for patients with advanced/metastatic CRC by pooling the currently available clinical trial data. While the treatment efficacy was heterogeneous across the trials, the microsatellite phenotype and drug regimen were the primary factors influencing the treatment response. While most regimens showed low treatment efficacy for MSS/pMMR CRC, a durable response was noted in the second- or more-line treatment when ICI was administered as part of a combination treatment, which suggests the potential of ICI treatment for MSS/pMMR CRCs and indicates the need for further research. 

## Figures and Tables

**Table 1:** Characteristics of the studies included in the meta-analysis.

**Author (Year)**	**Phase**	Sample Size	Disease State	Drug Regimen	Treatment Line	Microsatellite Phenotype	ORR ^1^	DCR ^1^
Brahmer JR et al. (2010) [28]	I	14	Metastatic CRC	Nivolumab	2L+	NR	1 (7.1)	NR
Brahmer JR et al. (2012) [29]	I	18	Advanced/metastatic CRC	Nivolumab	2L+	NR	0 (0)	NR
Topalian SL et al. (2012) [44]	I	19	Advanced CRC	Nivolumab	2L+	NR	0 (0)	NR
Wallin J et al. (2016) [45]	I	23	Metastatic CRC	Atezolizumab + BEV + FOLFOX	1L	NR	12 (0.5)	NR
Segal NH et al. (2016) [40]	II	26	Metastatic CRC	Pembrolizumab + (RT or RFA)	3L+	MSS/pMMR	1 (3.8)	NR
Overman MJ et al. (2017) [5]	II	74	Metastatic CRC	Nivolumab	2L+	MSI-H/dMMR	23 (31.1)	51 (68.9)
Shahda S et al. (2017) [42]	II	30	Advanced CRC	Pembrolizumab + mFOLFOX6	1L	NR	16 (53.3)	30 (100)
Lee JJ et al. (2017) [36]	II	30	Metastatic CRC	Pembrolizumab + Azacitidine	2L+	MSS/pMMR	1 (3.3)	4 (13.3)
O’Neil et al. (2017) [38]	I	23	Advanced/metastatic CRC	Pembrolizumab	2L+	22 MSS/pMMR, 1 MSI-H/dMMR	1 (4.3)	5 (21.7)
		22		Pembrolizumab	2L+	MSS/pMMR	0 (0)	4 (18.2)
		1		Pembrolizumab	2L+	MSI-H/dMMR	1 (100)	1 (100)
Segal NH et al. (2017) [41]	I	14	Metastatic CRC	Atezolizumab + CEA-CD3 TCB	2L+	NR	3 (21.4)	8 (57.1)
Overman MJ et al. (2018) [6]	II	119	Recurrent/metastatic CRC	Nivolumab + Ipilimumab	2L+	MSI-H/dMMR	65 (54.6)	102 (85.7)
Eng C et al. (2019) [31]	III	273	Advanced/metastatic CRC	Atezolizumab mono or combined with cobimetinib	3L+	NR	7 (2.6)	67 (24.5)
		183		Atezolizumab + Cobimetinib	3L+	NR	5 (2.7)	48 (26.2)
		90		Atezolizumab	3L+	NR	2 (2.2)	19 (21.1)
Floudas CS et al. (2019) [32]	I	15	Metastatic CRC	AMP-224 (anti pd-1) + Cy + RT	2L+	NR	0 (0)	3 (20.0)
Hellmann MD et al. (2019) [33]	I	84	Metastatic CRC	Atezolizumab + Cobimetinib	2L+	NR	7 (8.3)	26 (31.0)
Mettu NB et al. (2019) [37]	II	82	Metastatic CRC	Capecitabine + BEV + Atezolizumab	2L+	mostly MSS/pMMR (85.7%)	7 (8.5)	NR
Cousin S et al. (2019) [17]	II	48	Metastatic CRC	Avelumab + Regorafenib	2L+	MSS/pMMR	0 (0)	23 (47.9)
Parikh AR et al. (2019) [39]	II	40	Metastatic CRC	Nivolumab + Ipilimumab + RT	3L+	MSS/pMMR	3 (7.5)	7 (17.5)
André T et al. (2020) [7]	III	153	Metastatic CRC	Pembrolizumab	1L	MSI-H/dMMR	67 (43.8)	99 (64.7)
Cohen R et al. (2020) [30]	II	57	Metastatic CRC	Nivolumab + Ipilimumab	2L+	MSI-H/dMMR	34 (59.6)	51 (89.5)
Fukuoka S et al. (2020) [14]	I	25	Advanced/metastatic CRC	Nivolumab + Rigorafenib	3L+	mostly MSS/pMMR	9 (36.0)	21 (84.0)
		24		Nivolumab + Rigorafenib	3L+	MSS/pMMR	8 (33.3)	20 (83.3)
		1		Nivolumab + Rigorafenib	3L+	MSI-H/dMMR	1 (100)	0 (0)
Kawazoe A et al. (2020) [15]	II	50	Metastatic CRC	Pembrolizumab + Napabucasin	2L+	MSI-H/dMMR or MSS/pMMR	9 (18.0)	27 (54.0)
		40	Metastatic CRC	Pembrolizumab + Napabucasin	2L+	MSS/pMMR	4 (10.0)	18 (45.0)
		10	Metastatic CRC	Pembrolizumab + Napabucasin	2L+	MSI-H/dMMR	5 (50.0)	9 (90.0)
Kim JH et al. (2020) [34]	II	33	Metastatic CRC	Avelumab	2L+	MSI-H/dMMR or POLE mutations	8 (24.2)	26 (78.8)
		21	Metastatic CRC	Avelumab	2L+	MSI-H/dMMR	6 (28.6)	19 (90.5)
		3	Metastatic CRC	Avelumab	2L+	pole	0 (0)	0 (0)
Le DT et al. (2020) [35]	II	124	Advanced/metastatic CRC	Pembrolizumab	2L+	MSI-H/dMMR	41 (33.1)	67 (54.0)
		61		Pembrolizumab	3L+	MSI-H/dMMR	20 (32.8)	31 (50.8)
		63		Pembrolizumab	2L+	MSI-H/dMMR	21 (33.3)	36 (57.1)
Taylor K et al. (2020) [43]	II	15	Metastatic CRC	CC-486 + Durvalumab	4L+	MSS/pMMR	0 (0)	NR
Yarchoan M et al. (2020) [46]	II	17	Metastatic CRC	GVAX + Cy + Pembrolizumab	3L+	MSS/pMMR	0 (0)	3 (17.6)
NCT01876511 (2020) ^2^	II	66	Metastatic CRC	Pembrolizumab	2L+	MSI-H/dMMR or MSS/pMMR		
		41		Pembrolizumab	2L+	MSI-H/dMMR	22 (54.0)	33 (80.0)
		25		Pembrolizumab	2L+	MSS/pMMR	0 (0)	4 (16.0)
Grothey A et al. (2018) [8]	II	297	Metastatic CRC	FOLFOX + BEV + Atezolizumab	1L	NR	49 (16.5)	227 (76.4)
Lenz HJ et al. (2020) [9]	II	45	Metastatic CRC	Nivolumab + Ipilimumab	1L	MSI-H/dMMR	31 (68.9)	38 (84.4)
Stein A et al. (2020) [10]	II	39	Metastatic CRC	Avelumab + mFOLFOX6 + Cetuximab	1L	NR	31 (79.5)	36 (92.3)
Kim R et al. (2020) [16]	I	17	Metastatic CRC	Nivolumab + Regorafenib	2L+	MSS/pMMR	10 (58.8)	10 (58.8)

^1^ Data are number with percentage in parentheses. ^2^ Available from clinicaltrials.gov/ct2/show/NCT01876511 (accessed on 17 January 2021). CRC, colorectal cancer; DCR, disease control rate; ORR, overall response rate.

**Table 2:** The Newcastle–Ottawa scale quality assessment for the 25 non-randomized clinical trials.

**Study**	Selection				Comparability	Outcome			Total ^1^
	Representativeness of the Exposed Cohort	Selection of the Non-Exposed Cohort	Ascertainment of Exposure	Demonstration That Outcome of Interest Was Not present at the Start of the Study	Comparability on the Basis of Design and Analysis	Ascertainment of Outcome	Adequate Follow-Up	Adequacy of Follow-Up of Cohorts	
Brahmer JR et al. (2010) [28]	1	1	1	1	2	1	0	0	7
Brahmer JR et al. (2012) [29]	1	1	1	1	2	1	0	0	7
Topalian SL et al. (2012) [44]	1	1	1	1	2	1	0	0	7
Segal NH et al. (2016) [40]	1	1	1	1	2	1	0	0	7
Overman MJ et al. (2017) [5]	1	1	1	1	2	1	1	1	9
Shahda S et al. (2017) [42]	1	1	1	1	2	1	1	1	9
Lee JJ et al. (2017) [36]	1	1	1	1	2	1	0	0	7
O’Neil et al. (2017) [38]	1	1	1	1	2	1	1	1	9
Segal NH et al. (2017) [41]	1	1	1	1	2	1	0	0	7
Overman MJ et al. (2018) [6]	1	1	1	1	2	1	1	1	9
Floudas CS et al. (2019) [32]	1	1	1	1	2	1	0	0	7
Hellmann MD et al. (2019) [33]	1	1	1	1	2	1	0	0	7
Cousin S et al. (2019) [17]	1	1	1	1	2	1	1	1	9
Parikh AR et al. (2019) [39]	1	1	1	1	2	1	0	0	7
Cohen R et al. (2020) [30]	1	1	1	1	2	1	1	1	9
Fukuoka S et al. (2020) [14]	1	1	1	1	2	1	1	1	9
Kawazoe A et al. (2020) [15]	1	1	1	1	2	1	0	0	7
Kim JH et al. (2020) [34]	1	1	1	1	2	1	1	1	9
Le DT et al. (2020) [35]	1	1	1	1	2	1	0	0	7
Taylor K et al. (2020) [43]	1	1	1	1	2	1	0	0	7
Yarchoan M et al. (2020) [46]	1	1	1	1	2	1	0	0	7
NCT01876511 (2020)	1	1	1	1	2	1	0	0	7
Lenz HJ et al. (2020) [9]	1	1	1	1	2	1	1	1	9
Stein A et al. (2020) [10]	1	1	1	1	2	1	0	0	7
Kim R et al. (2020) [16]	1	1	1	1	2	1	1	1	9

^1^ Each study could be awarded a maximum of 9: a maximum of 2 for the item regarding comparability, and a maximum of 1 for the other seven items.

**Table 3:** Results of the univariate and multivariate meta-regression analyses.

**Treatment Efficacy**	Parameter	Pooled Estimate (%)	Univariate Analysis		Multivariate Analysis	
			Odds Ratio	*p* Value	Odds Ratio	*p* Value
ORR	Treatment line			0.003		0.394
	First-line	51.5 (29.2–73.6)	Reference		Reference	
	Second- or more-line	13.4 (6.4–22.2)	0.67 (0.52–0.86)		0.90 (0.71–1.15)	
	Microsatellite phenotype			<0.001		<0.001
	MSS/pMMR	5.9 (0.6–14.6)	Reference		Reference	
	MSI-H/dMMR	46.8 (37.9–55.9)	1.61 (1.37–1.90)		1.67 (1.42–1.98)	
	Drug regimen			0.332		0.019
	ICI monotherapy	14.2 (5.3–26.0)	Reference		Reference	
	ICI combination therapy	22.4 (11.8–35.0)	1.11 (0.89–1.39)		1.21 (1.04–1.42)	
DCR	Treatment line			0.006		0.613
	First-line	85.0 (72.6–94.3)	Reference		Reference	
	Second- or more-line	49.5 (36.2–62.8)	0.68 (0.52–0.88)		1.07 (0.81–1.41)	
	Microsatellite phenotype			<0.001		<0.001
	MSS/pMMR	34.2 (19.4–50.6)	Reference		Reference	
	MSI-H/dMMR	78.4 (68.6–86.9)	1.57 (1.29–1.91)		1.72 (1.41–2.10)	
	Drug regimen			0.632		0.032
	ICI monotherapy	52.4 (37.1–67.5)	Reference		Reference	
	ICI combination therapy	58.7 (42.9–73.7)	1.07 (0.81–1.39)		1.24 (1.02–1.49)	

Numbers in parenthesis are 95% confidence intervals. DCR, disease control rate; ICI, immune checkpoint inhibitor; MSI-H, microsatellite instability-high; MSS, microsatellite instability-stable; ORR, overall response rate.

**Table 4:** Subgroup analyses according to the microsatellite phenotype and drug regimen in patients treated with ICIs as second- or more-line therapy.

**Treatment Efficacy**	Category	Pooled Estimate (%)	*p* Value			
			vs. Category 1	vs. Category 2	vs. Category 3	vs. Category 4
ORR	Category 1 (mono, MSI-H)	36.1 (26.7–46.1)	-	<0.001	0.017	<0.001
	Category 2 (mono, MSS)	0.0 (0.5–4.1)	<0.001	-	<0.001	0.318
	Category 3 (combi, MSI-H)	56.1 (48.6–63.4)	0.017	<0.001	-	<0.001
	Category 4 (combi, MSS)	8.3 (1.2–19.3)	<0.001	0.318	< 0.001	-
DCR	Category 1 (mono, MSI-H)	72.8 (56.9–86.3)	-	0.001	0.320	0.011
	Category 2 (mono, MSS)	17.0 (7.1–29.5)	0.001	-	<0.001	0.142
	Category 3 (combi, MSI-H)	88.0 (82.6–92.7)	0.320	<0.001	-	<0.001
	Category 4 (combi, MSS)	40.8 (21.6–61.5)	0.011	0.142	<0.001	-

DCR, disease control rate; ORR, overall response rate.

## Data Availability

Data sharing is not applicable to this article as no new data were created or analyzed in this study.

## Supplementary Materials

The following are available online at https://www.mdpi.com/article/10.3390/jcm10163599/s1, Figure S1: Funnel plot for the overall response rate, Figure S2: Funnel plot for the disease control rate.
