# Supplementary material for: Treatment Efficacy of Immune Checkpoint Inhibitors for Patients with Advanced or Metastatic Colorectal Cancer: A Systematic Review and Meta-Analysis
Source: J Clin Med. 2021 Aug 16;10(16):3599. doi: 10.3390/jcm10163599 (PMC8397178; doi:10.3390/jcm10163599)
Supplement: Supplementary file 1 [file jcm-10-03599-s001.zip › jcm-1286035-supplementary.pdf]

Supplementary Figures

Figure S1. Funnel plot for the overall response rate.

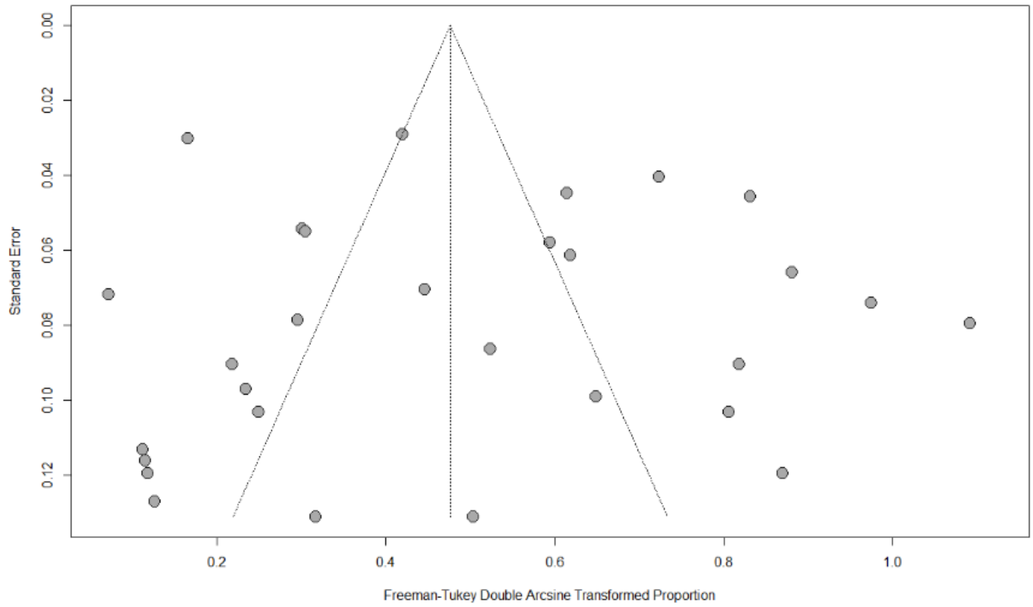

The funnel plot generated to visually appraise bias in the literature for the overall response rate (proportion of patients who achieved complete or partial response according to RECIST v1.1) revealed no substantial publication bias.

Figure S2. Funnel plot for the disease control rate.

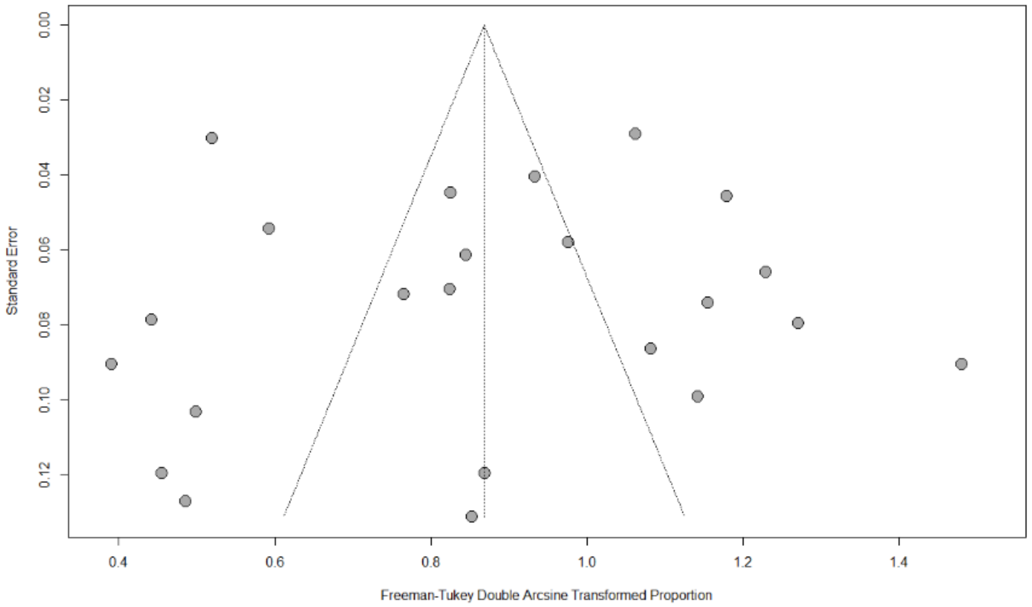

The funnel plot generated to visually appraise bias in the literature for the disease control rate (proportion of patients who achieved complete response, partial response, and stable disease according to RECIST v1.1) revealed no substantial publication bias.
